# Supplementary material for: The contribution of short tandem repeats to splicing variation in the human cortex
Source: bioRxiv. 2026 May 6:2026.05.04.721407. Preprint. [Version 1] doi: 10.64898/2026.05.04.721407 (PMC13174678; doi:10.64898/2026.05.04.721407)
Supplement: Supplement 2 [file media-2.pdf]

Supplementary Figures

Supplementary Figure 1

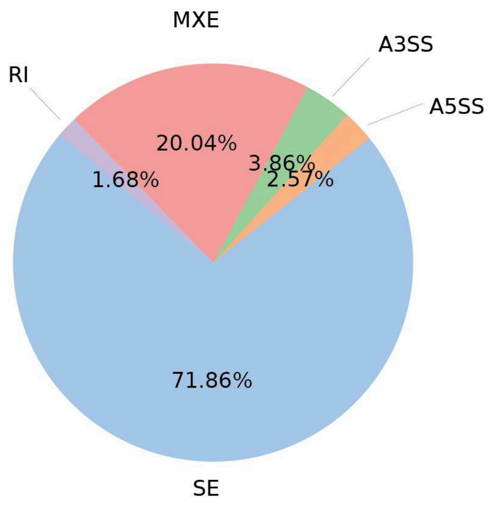

Summary of splice event types identified by rMATs in the HBCC dataset. A total of 548,614 splice events were identified by rMATs. The pie chart shows the percentage of the five alternative splicing patterns considered: skipped exon (SE, n=394,222), alternative 5' splice sites (A5SS, n=14,094), alternative 3' splice sites (A3SS, n=21,171), mutually exclusive exons (MXE, n=109,921), or retained intron (RI, n=9,206).

Supplementary Figure 2

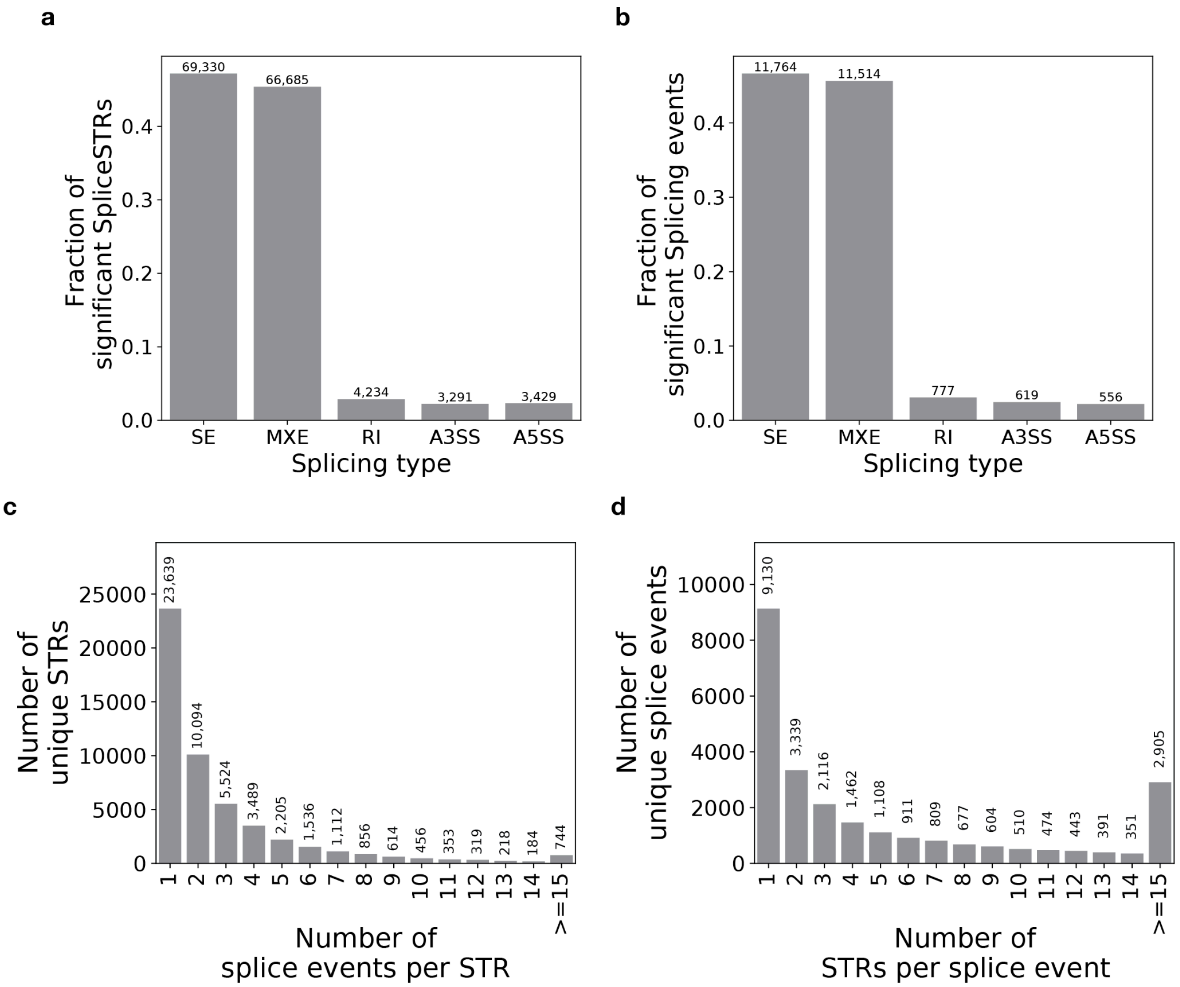

Summary of splicing event types across significant spliceSTRs. (a) The fraction of STR-splice event pairs tested that were identified as significant spliceSTRs (FDR 5%) stratified by splice event type. A total of 146,969 significant STR-splice event pairs were identified. **(b)** The fraction of tested splicing events identified as having at least one significant spliceSTR stratified by event type. A total of 25,230 unique splicing events had at least one spliceSTR. In each panel, the *x*-axis represents the type of splice event. Exact values are shown above each bar. **(c)** The number of unique splice events significantly associated per STR. A total of 51,343 unique significant STRs were identified. **(d)** The number of unique STRs significantly associated per splice event. A total of 25,230 unique significant splice events were identified. Bars represent counts of unique STRs or splice events (*y*-axis) across different categories (*x*-axis). Numerical values above each bar indicate the total count for that category. The median number of unique splice events per STR is 2 and the median number of unique STRs per splice event is 3.

Supplementary Figure 3

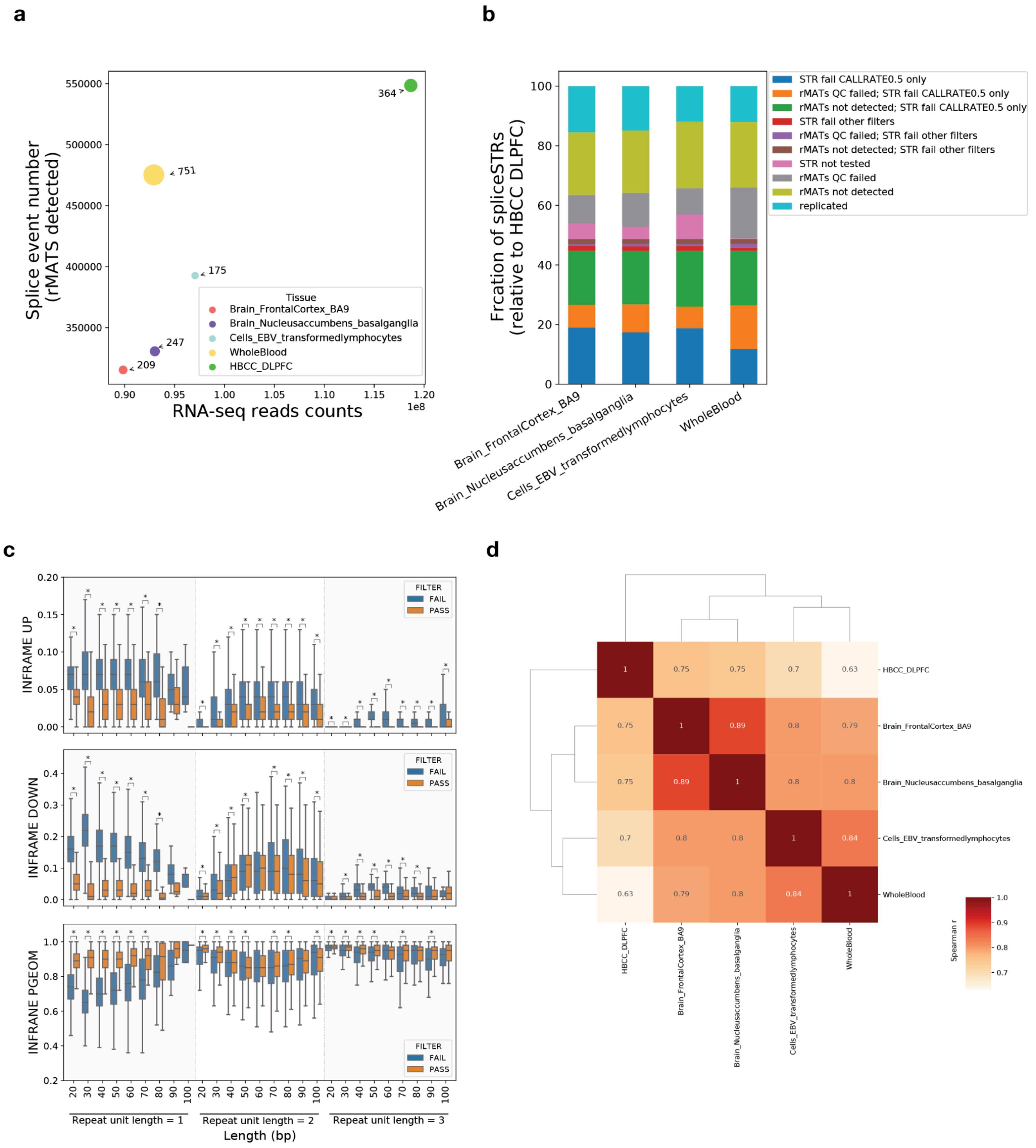

Comparison of spliceSTRs identified in the HBCC DLPFC with those found in four GTEx tissues. (a) Relationship between read counts from RNA-sequencing and number of splice events detected by rMATs. The *x*-axis shows the mean read counts of the RNA-sequencing across all samples in each tissue. The *y*-axis shows the total number of alternative splice events detected by rMATs across all samples per tissue. Each dot represents a single tissue as indicated by the legend. The size of the dot corresponds to the sample size of

each tissue. The numbers show the exact sample size of each tissue. Although HBCC DLPFC does not have the largest sample size, it has the deepest RNA-seq coverage and correspondingly the highest number of splice events detected by rMATs. **(b)** Categorization of spliceSTR events identified as significant in HBCC DLPFC that could be tested in each of the four GTEx tissues considered. The *x*-axis shows the name of the tissue from GTEx. The *y*-axis shows the percentage of significant spliceSTRs detected in HBCC DLPFC in each category. Cyan indicates spliceSTRs for which both the STR and splicing event passed QC filters in the GTEx dataset. Other colors indicate the percentage of spliceSTRs filtered due to failing either STR genotyping or rMATs QC steps. **(c)** Comparison of stutter model parameters between STRs that passed and failed HipSTR locus-level filters (**Methods**). Per-locus stutter parameters were estimated from PCR+ high-coverage WGS for GTEx samples using HipSTR. Box plots in each row show the distribution of the three stutter parameters output by HipSTR for each STR (top=INFRAME_UP, which denotes the estimated fraction of reads showing a PCR stutter error that increases repeat length; middle=INFRAME_DOWN, which denotes the estimated fraction of reads showing a PCR stutter error that decreases repeat length; and bottom=INFRAME_PGEOM, which denotes the fraction of stutter errors that result in changes of a single repeat unit). Results are stratified by repeat unit length. Horizontal lines show median values, boxes span from the 25th percentile (Q1) to the 75th percentile (Q3). Whiskers extend to $Q1 - 1.5 \times IQR$ (bottom) and $Q3 + 1.5 \times IQR$ (top), where IQR is the interquartile range (Q3–Q1). The *x*-axis in each panel shows bins of STR total length in base pairs. The color indicates "PASS" or "FAIL" by HipSTR filters (see **Methods**). The overall distributions of stutter parameters are similar to those observed in PCR+ high-coverage WGS samples from the H3Africa cohort[1]. Asterisks denote statistical significance (Mann–Whitney U test, nominal $P < 0.05$) between STRs that passed vs. failed HipSTR call filters. **(d)** Pairwise correlation of effects of significant spliceSTRs across tissues. Each cell shows the Pearson correlation between the effect sizes of spliceSTRs identified in either of the paired tissues at FDR 5%.

Supplementary Figure 4

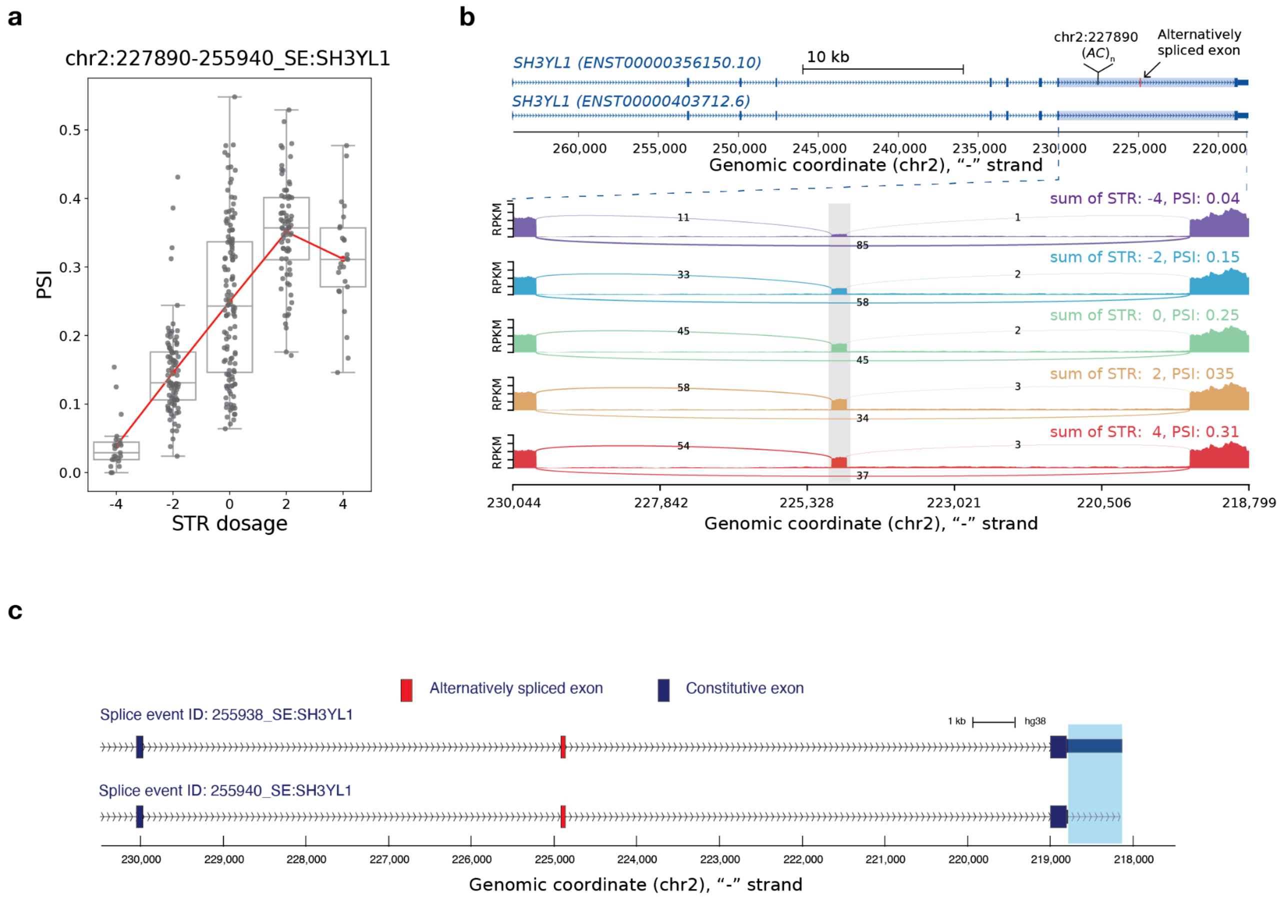

Example spliceSTR identified in the HBCC DLPFC that is also significant in three of four GTEx tissues tested. (a) The *x*-axis represents the STR dosage (sum of repeat lengths across both chromosome copies, in bp relative to the hg38 reference) of a STR (hg38:chr2:227890-227910) within an intron of *SH3YL1*. The *y*-axis represents the PSI of exon 9 (ENST00000356150.10). Each dot represents a single individual. Box plots summarize the distribution of PSI values. Horizontal lines show median values, boxes span from the 25th percentile (Q1) to the 75th percentile (Q3). Whiskers extend to Q1 − 1.5 × IQR (bottom) and Q3 + 1.5 × IQR (top), where IQR is the interquartile range (Q3–Q1). The red line shows the mean expression for each x-axis value. **(b)** Sashimi plot showing read-level evidence for the example spliceSTR. This spliceSTR has the same STR and associated exon as that shown in **Fig. 1d**, but was identified in GTEx with a different boundary of the downstream constitutive exon (see panel **c**). The full exonic structure of two *SH3YL1* transcripts and the location of the splicesSTR (hg38:chr2:227890-227910) are shown in the top panel. Samples were grouped by STR dosage. The alternatively spliced exon is marked by a grey box. To better visualize the spliced exon, intronic regions are displayed at one-fifth the scale of exonic regions. Coordinates are in descending order since annotations are shown for the reverse strand relative to hg38. Sashimi plots were generated using rmats2sashimiplot (v2.0.4). **(c) Exonic structure of the two similar splice events.** The exonic structure of splice event ID 255938_SE:SH3YL1 identified in HBCC from **Fig 1d** is shown in the top panel. A similar splice event with ID 255940_SE:SH3YL1 identified in both HBCC and GTEx is shown in the bottom panel. The boundary difference between the two splice events is highlighted with a blue box.

Supplementary Figure 5

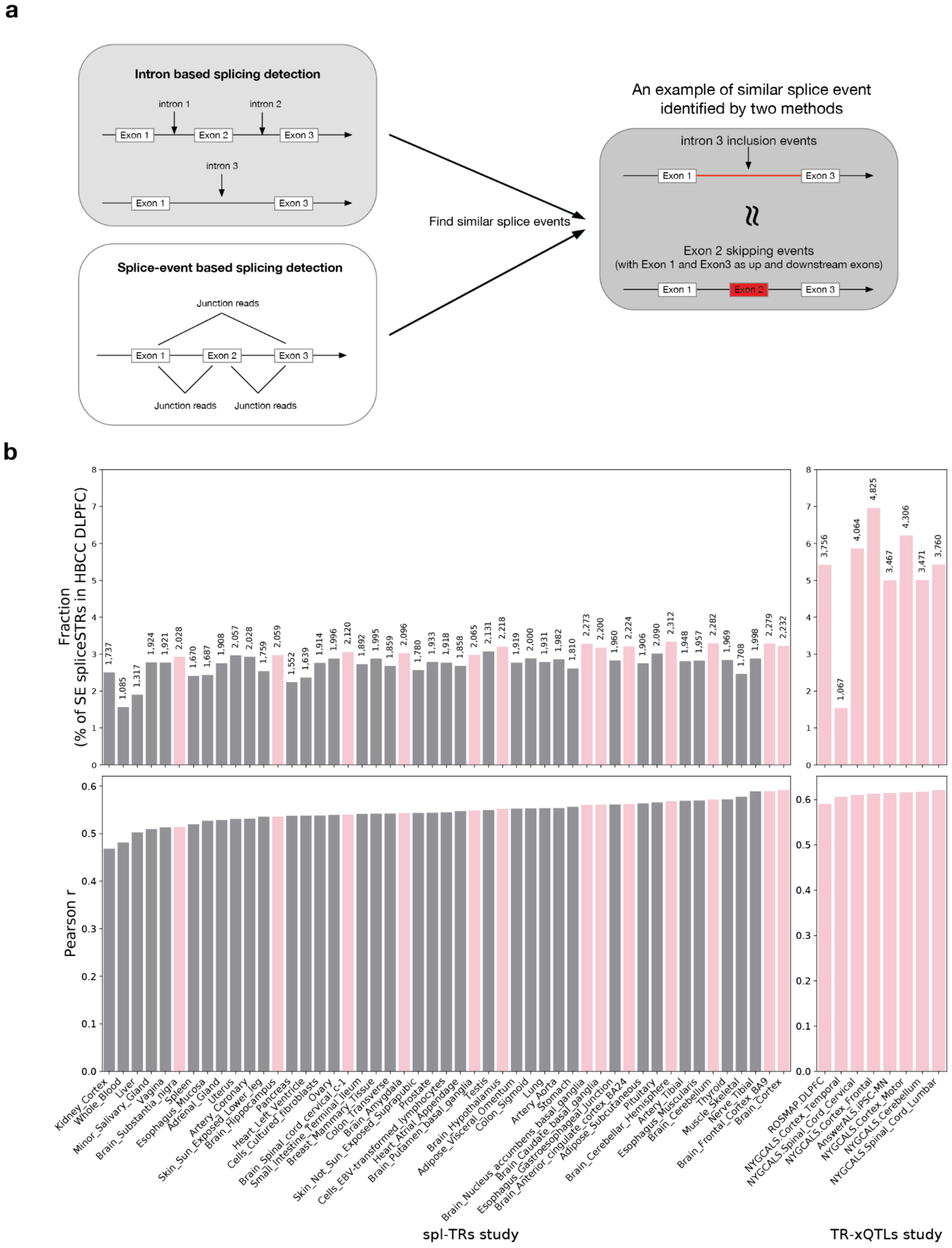

Comparison of spliceSTRs identified in HBCC DLPFC with those reported in other studies. (a) Schematic diagram shows the two distinct methods for detecting splice events. Intron-based detection measures the inclusion of an intron, whereas splice-event based detection assesses the inclusion of an exon. Our study used rMATs to perform splice-event based detection, whereas the studies compared to used intron-based detection. To enable comparison, we focused on skipped exon events identified by rMATS and

matched them to corresponding intron-based events by comparing intron lengths. Notably, exon inclusion corresponds to the exclusion of the intron between the two flanking constitutive exons. **(b)** Replication of HBCC DLPFC spliceSTRs vs. other studies (spl-TRs study[1,2], TR-xQTLs study[3]). The upper panel shows the fraction of significant skipped exon spliceSTRs identified in HBCC DLPFC that were also tested in each tissue from other studies. The number of overlapping events is labeled above each bar. The lower panel displays the Pearson correlation of effect sizes for shared skipped exon spliceSTRs between HBCC DLPFC and each tissue from the other studies. Tissue names from each study are shown on the *x*-axis. Pink and grey bars represent brain and non-brain tissues respectively.

Supplementary Figure 6

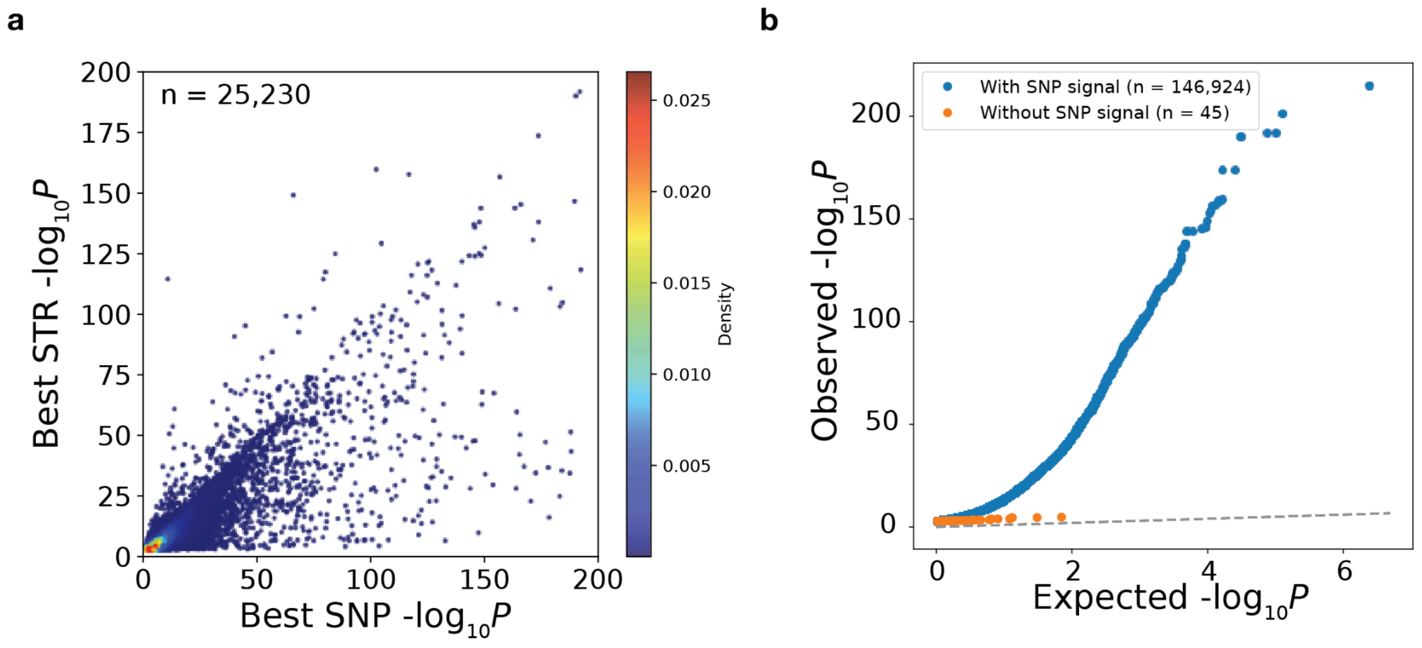

Comparison of P-values from SNP and STR splicing association tests. (a) Comparison of the best SNP vs. best STR P-value for each splice event. Color denotes the density of points in each region. **(b)** Comparison of *P*-values between spliceSTRs with or without SNP signals detected. The quantile-quantile plot compares the observed $-\log_{10}$ *P*-values (*y*-axis) vs. the expected $-\log_{10}$ *P*-values (*x*-axis) for spliceSTRs. SpliceSTRs were grouped by whether their associated splice events also exhibit significant SNP signals (blue=events that also have a SNP signal, orange=events that only have an STR signal). The x-axis represents the expected uniform distribution of $-\log_{10}$ *P*-values for each group of spliceSTRs. A total of 45 spliceSTRs were associated with 44 unique splicing events for which no SNP signal was detected.

Supplementary Figure 7

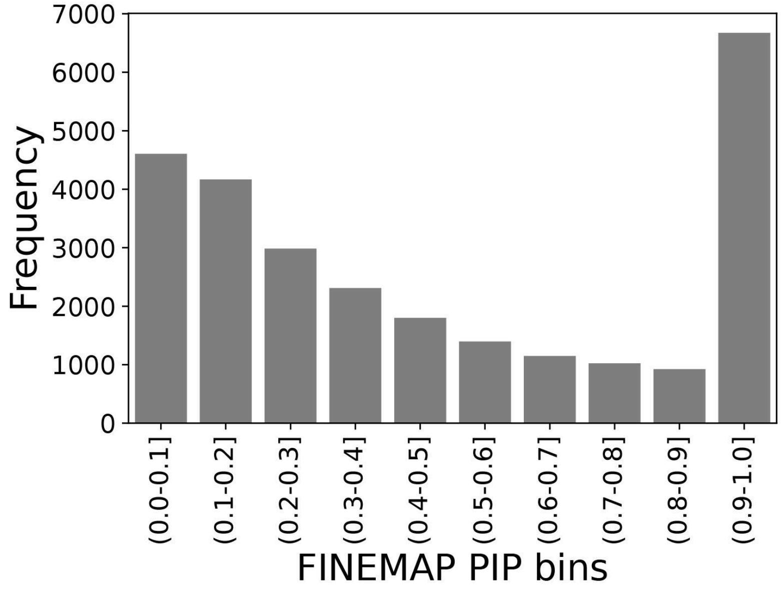

Distribution of the top FINEMAP PIPs across all splice events. The *x*-axis represents the bins of top PIPs for any variant (SNP or STR) for each splice event across all events tested. For 64.60% (n =15,866) of splicing events analyzed using FINEMAP, no single variant (SNP or STR) reached a threshold of PIP>0.5.

Supplementary Figure 8

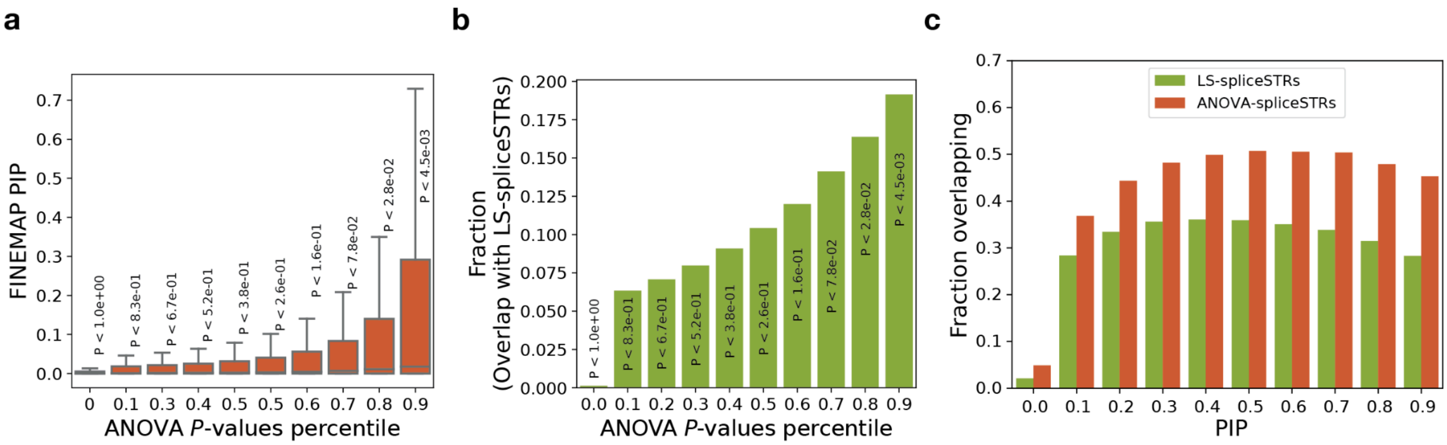

Concordance of fine-mapping results across methods. **(a)** Distribution of FINEMAP PIPs for spliceSTRs thresholded by ANOVA *P*-values. The *x*-axis represents deciles of ANOVA *P*-values (from weaker to stronger). The *y*-axis represents FINEMAP PIPs for spliceSTRs with ANOVA *P*-values below the threshold used to define each bin. Thresholds for each bin are labeled on top of each box. **(b)** Fraction of spliceSTRs that are LS-spliceSTRs thresholded by ANOVA *P*-values. The *y*-axis gives the fraction of spliceSTRs with ANOVA *P*-values below the threshold used to define each bin that are also LS-spliceSTRs. **(c)** Overlap between FM-spliceSTRs and candidate spliceSTRs identified by Lasso or ANOVA. The *x*-axis represents FINEMAP PIP thresholds used to group spliceSTRs. The *y*-axis represents the fraction of spliceSTRs with PIP above the thresholds and passing each alternative method (green=LASSO, red=ANOVA).

Supplementary Figure 9

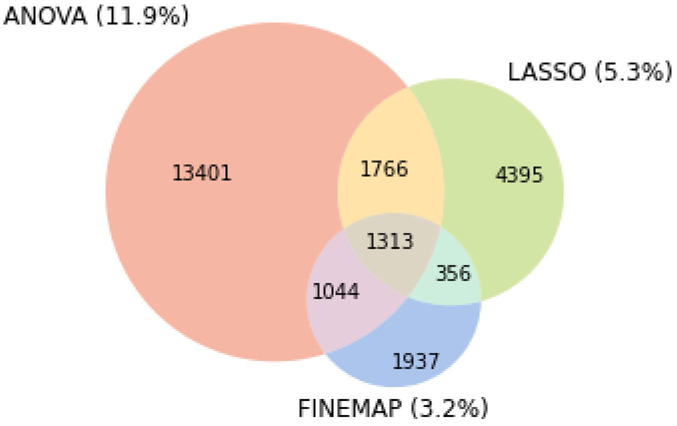

Overlapping of fine-mapped spliceSTRs across methods. Venn diagram showing the number of overlap between three fine-mapping approaches: ANOVA (pink), LASSO (green), and FINEMAP (blue). The percentage in parentheses indicates the fraction of spliceSTRs (out of all spliceSTRs passing FDR 5%) passing each method (ANOVA: 11.9%, LASSO: 5.3%, FINEMAP: 3.2%).

Supplementary Figure 10

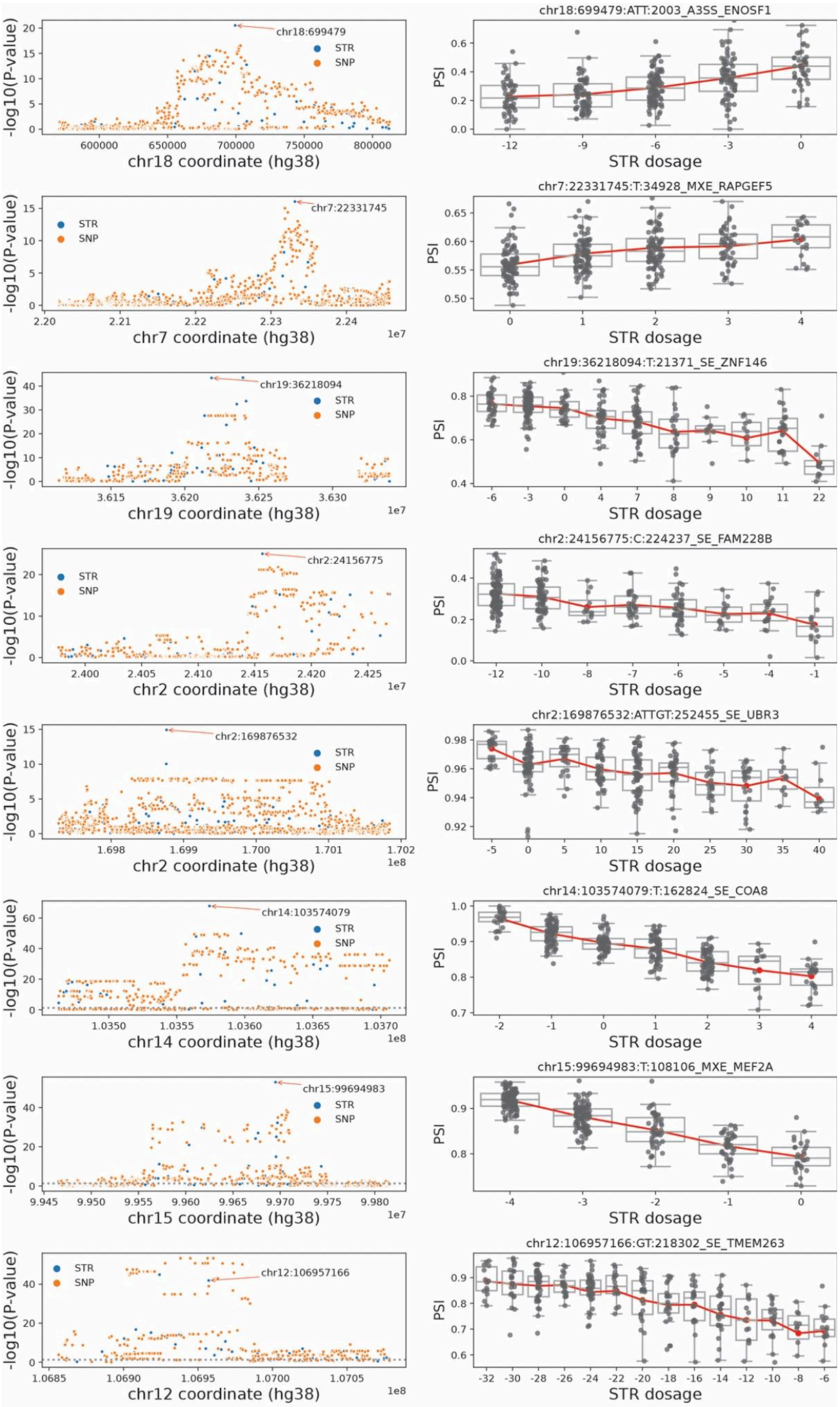

Additional examples of fine-mapped spliceSTRs. Each row shows a different spliceSTR for which at least two fine-mapping methods identified a candidate causal STR. Left panels show associations of all tested variants with PSI of the target splice event (blue=STRs, orange=SNPs). The candidate STR is indicated with a red arrow. Genomic coordinates are based on the hg38 reference assembly. Right panels show STR dosage (*x*-axis) vs. PSI (*y*-axis) for the target event. Note the *x*-axis ticks are evenly spaced and are in some cases not proportionally spaced according to STR dosage values.

Supplementary Figure 11

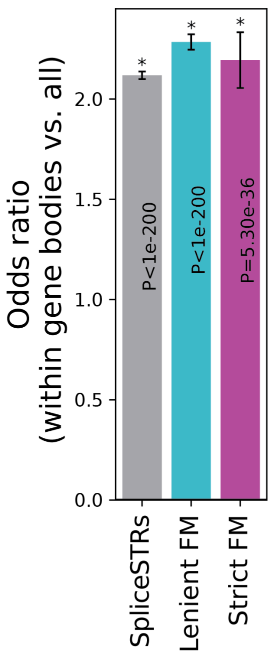

Fine-mapped spliceSTRs are enriched within gene transcription regions. The *y*-axis denotes the odds ratios comparing spliceSTRs or fine-mapped spliceSTRs within gene bodies to all tested STRs in each category. The *x*-axis represents the category of spliceSTR (grey=all spliceSTRs, cyan=lenient fine-mapped spliceSTRs, purple=strictly fine-mapped spliceSTRs). Error bars represent ± 1 s.e. Asterisks indicate statistically significant enrichment or depletion (two-tailed Fisher's exact test, Bonferroni corrected *P*<0.05) for each spliceSTR category.

Supplementary Figure 12

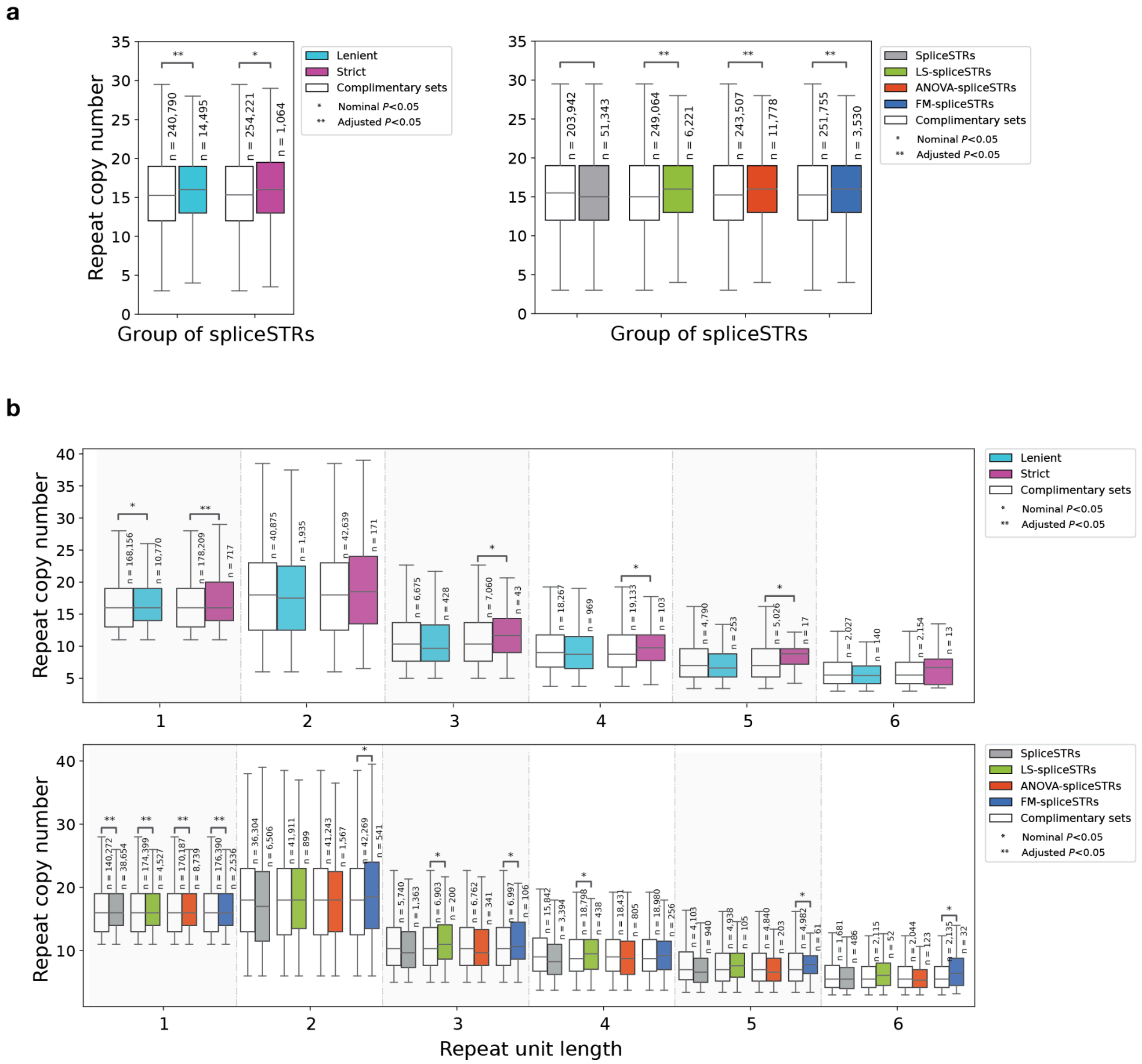

Comparison of repeat copy numbers between fine-mapped and non–fine-mapped spliceSTRs across different repeat unit lengths and fine-mapping methods. (a) Comparison of repeat copy numbers between fine-mapped and non–fine-mapped spliceSTRs across different fine-mapping methods. The *y*-axis shows the distribution of repeat copy numbers (based on hg38) within each group. **(b)** Comparison of repeat copy numbers stratified by repeat unit length. The *x*-axis represents the repeat unit length. The *y*-axis shows the distribution of repeat copy numbers (based on hg38) within each group. Colors indicate different fine-mapping methods, with “Complementary sets” in white denoting all tested spliceSTRs that were not fine-mapped using the method being compared to. Alternating shaded backgrounds separate repeat unit length categories. Asterisks denote statistical significance (two-sided Mann–Whitney U test) between each pair of distributions.

Supplementary Figure 13

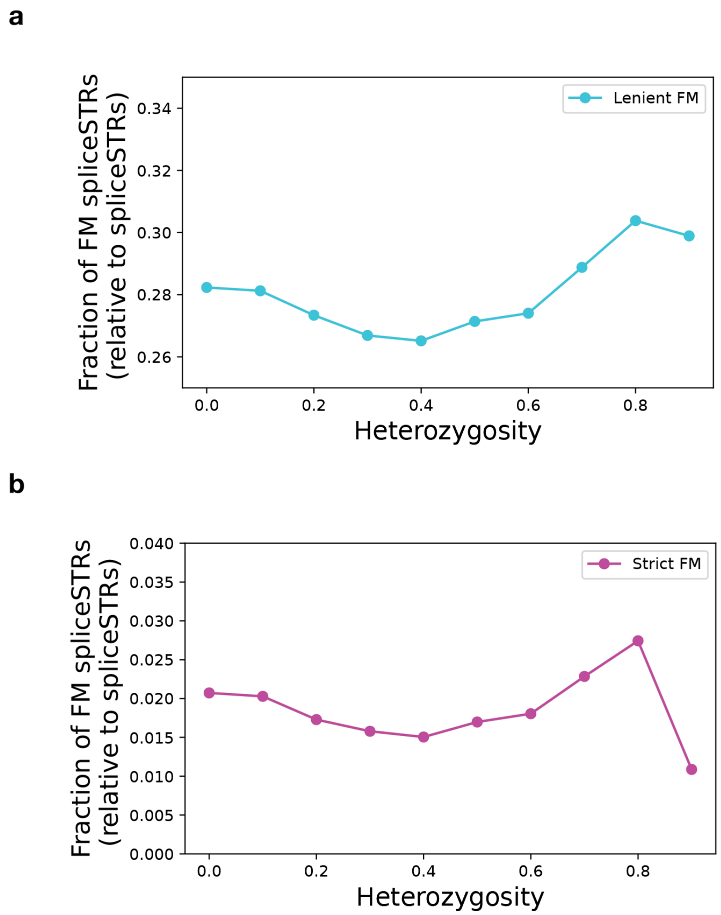

Fraction of fine-mapped spliceSTRs across heterozygosity thresholds. The *x*-axis denotes the minimum heterozygosity threshold to include an STR in our analysis. The *y*-axis shows the fraction of all STRs above that heterozygosity threshold that were significantly associated with at least one splice event that are also fine-mapped spliceSTRs (**a**: leniently fine-mapped spliceSTRs; **b**: strictly fine-mapped spliceSTRs).

Supplementary Figure 14

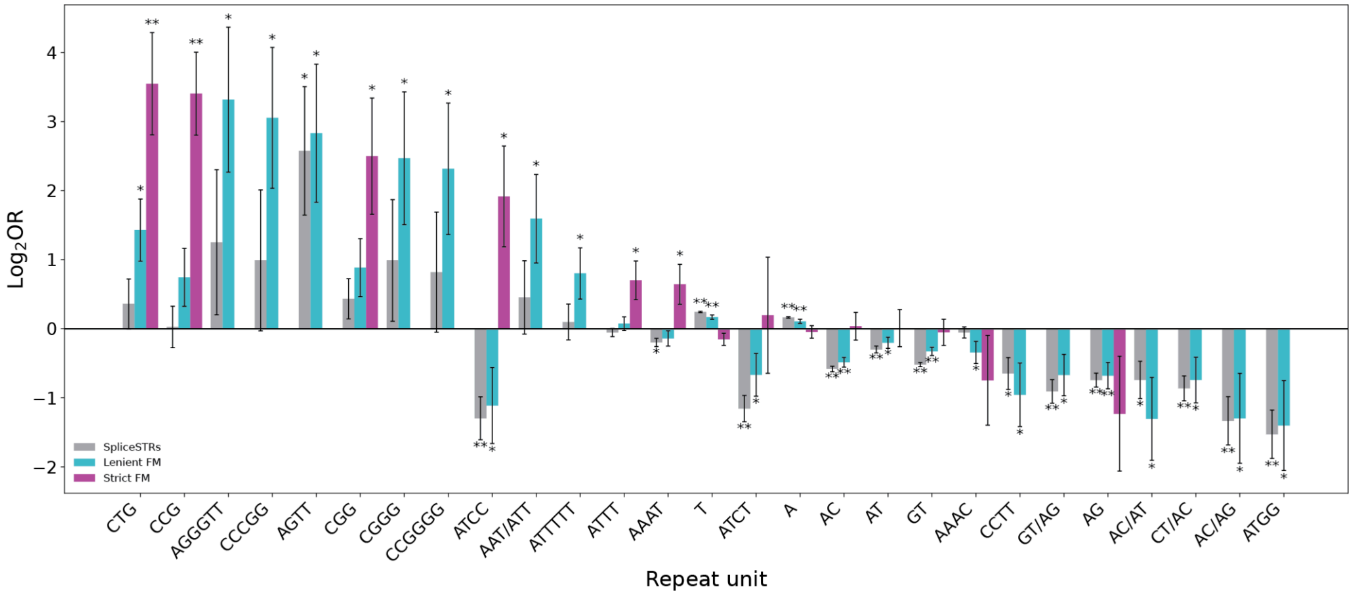

Comparison of repeat unit enrichment across different spliceSTR categories. The *x*-axis shows all repeat units for which there are at least 3 unique spliceSTRs for the corresponding spliceSTR group and the unit was significantly enriched. The *y*-axis denotes the $\log_2$ odds ratios comparing spliceSTRs or fine-mapped spliceSTRs to all tested STRs in each category. Error bars represent ± 1 s.e. Asterisks denote repeat units that are significantly enriched or depleted in each spliceSTR category (based on two-tailed Fisher exact *P*-value). Bars are colored by the spliceSTR category (gray=spliceSTRs, cyan=leniently fine-mapped spliceSTRs, purple=strictly fine-mapped spliceSTRs).

Supplementary Figure 15

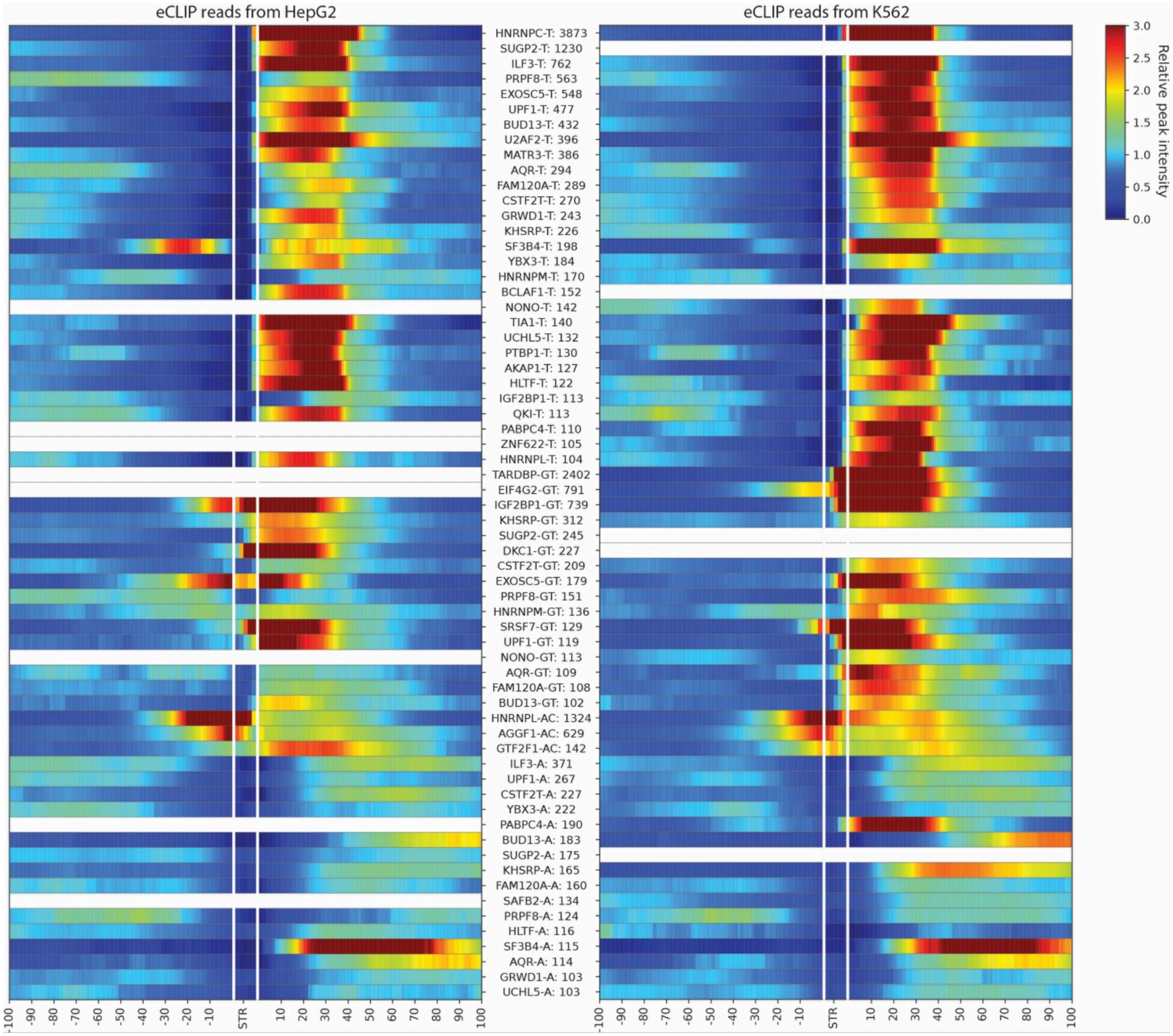

Heatmap plot showing aggregated RBP binding profiles overlapping tested STRs stratified by repeat unit in HepG2 and K562 cell lines. The *x*-axis represents the strand-specific distance from RNA-binding protein (RBP) binding peaks to their overlapping STR, with the STR centered at position zero. Negative values indicate upstream positions, while positive values indicate downstream. The *y*-axis lists RBP–repeat unit pairs along with the total number of overlapping repeat loci used in the analysis. Each row displays the aggregated RBP binding signal derived from the corresponding bigWig file. For each RBP–repeat unit pair, binding signals at individual loci were first normalized by the average signal of that locus, and the mean normalized signal across all loci was then used for plotting.

Supplementary Figure 16

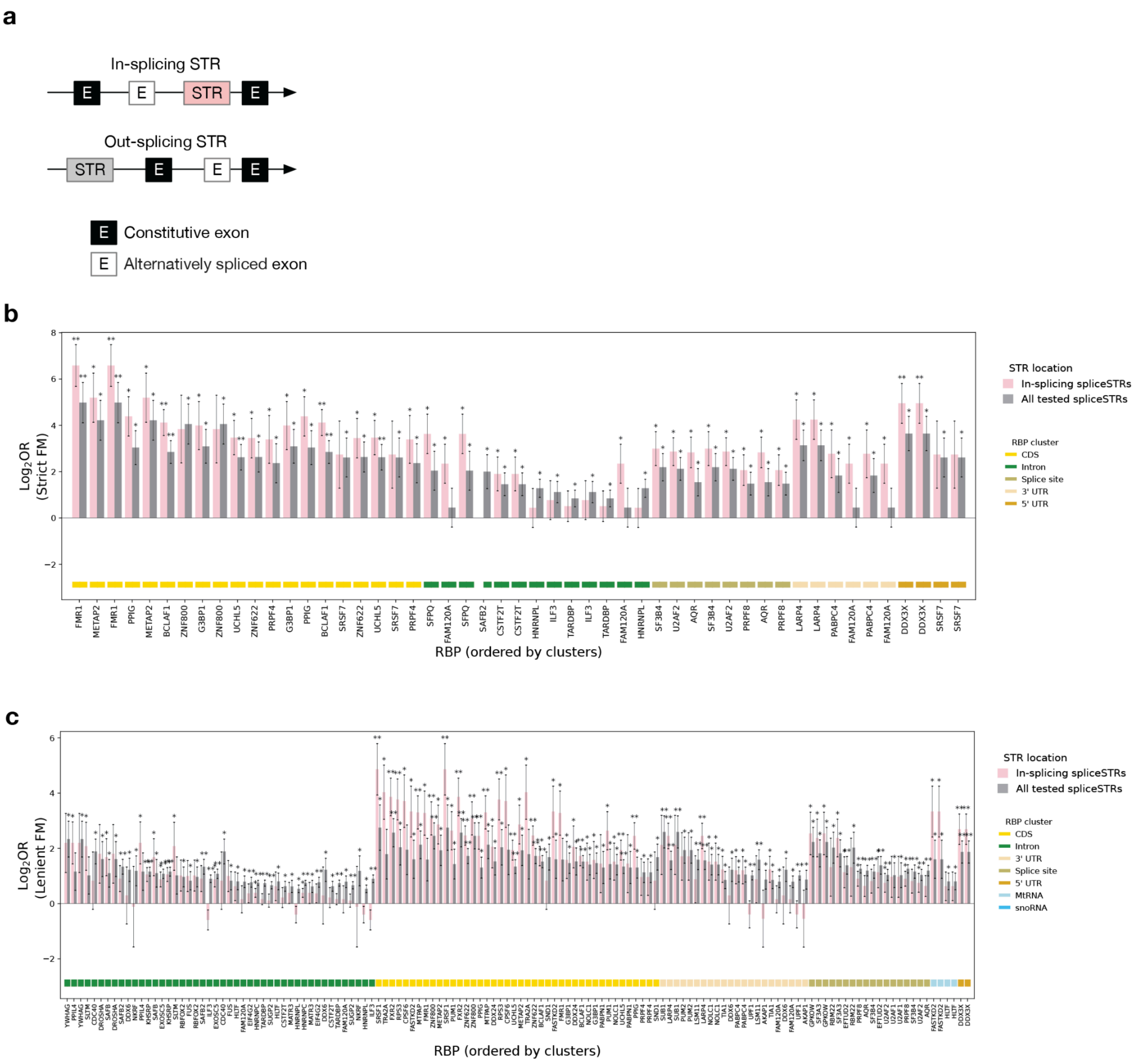

Comparison of enrichment for overlapping of RBP binding sites stratified by the location of spliceSTR in two fine-mapped spliceSTR sets. (a) Schematic defining spliceSTRs by their position relative to associated splice events. **(b)** Comparison of RBP binding sites enrichment between in-splicing and out-splicing STRs for leniently and **(c)** strictly fine-mapped spliceSTRs. Compared to all tested STRs, RBP enrichments are overall higher when we restrict analysis to STRs that are located within the constitutive exons of the splicing events. Compared to the leniently fine-mapped set, this trend is stronger for the strictly fine-mapped spliceSTRs. The x-axis shows all tested RBPs whose binding sites overlap at least three unique spliceSTRs from the corresponding fine-mapping group, as defined by Boyle et al.[4]. The y-axis represents the $\log_2$ odds ratios comparing fine-mapped spliceSTRs with all tested STRs within each STR location category for each RBP. Error bars indicate ±1 s.e. Asterisks denote RBPs for which binding sites are significantly enriched or depleted in each spliceSTR category (two-tailed Fisher's exact test, * indicates nominal $P<0.05$, ** indicates adjusted $P<0.05$). Bars are colored by spliceSTR location category (gray=all tested spliceSTRs; pink=in-splicing spliceSTRs).

Supplementary Figure 17

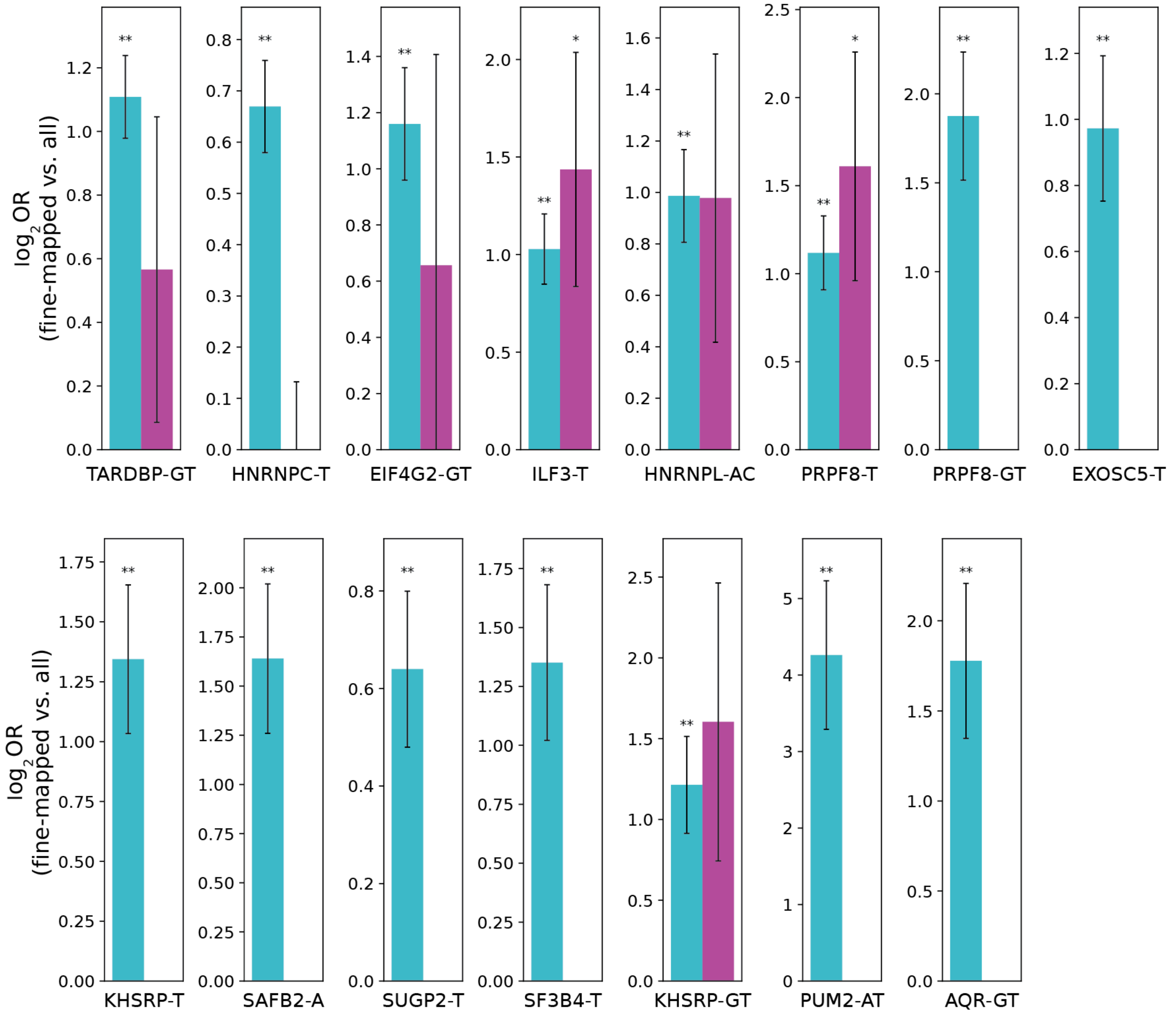

Additional examples of enriched repeat unit-RBP pairs among fine-mapped spliceSTRs. For each panel, the x-axis represents RBP-repeat unit pairs for which binding sites overlap at least 3 unique leniently fine-mapped spliceSTRs obtained from Boyle et al.[4]. The y-axis denotes the $\log_2$ odds ratio comparing each fine-mapped spliceSTR set (cyan=leniently fine-mapped spliceSTRs, purple=strictly fine-mapped spliceSTRs) to all tested STRs in each repeat unit-RBP pair. Error bars represent ± 1 s.e. Asterisks indicate statistically significant enrichment or depletion (two-tailed Fisher's exact test, * indicates nominal P<0.05, ** indicates adjusted P<0.05) for each group.

Supplementary Figure 18

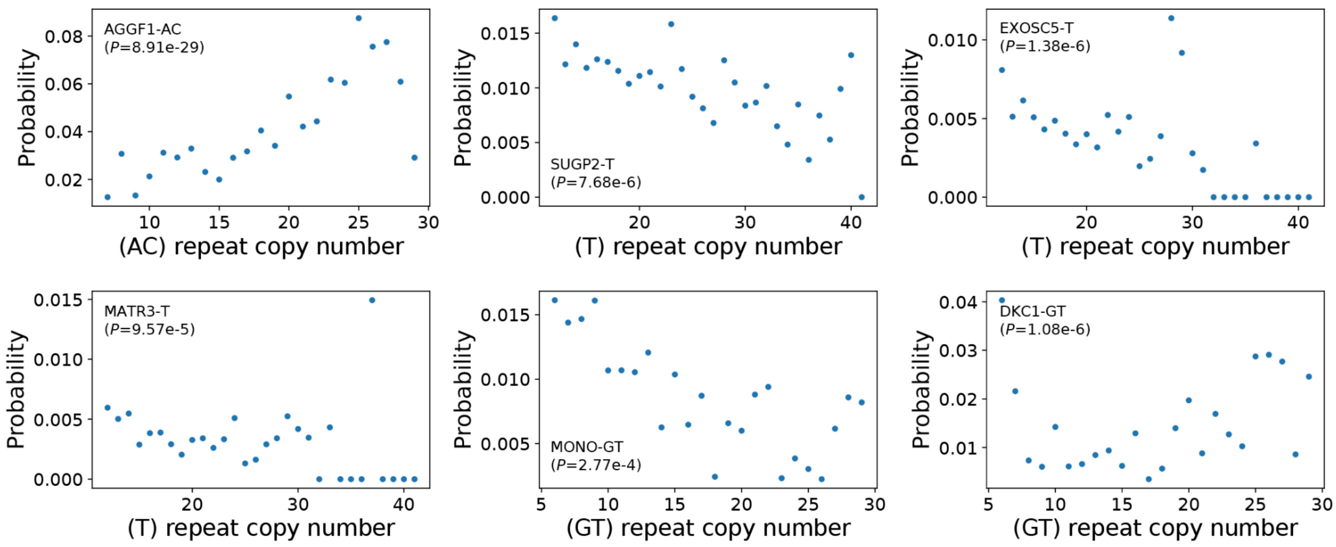

Additional examples of significant associations between RBP binding probability and repeat unit length. The *x*-axis represents the repeat unit copy number. The *y*-axis shows the probability of RBP binding across all STRs genome-wide of that length. *P*-values from the logistic regression are labeled in each plot.

Supplementary Figure 19

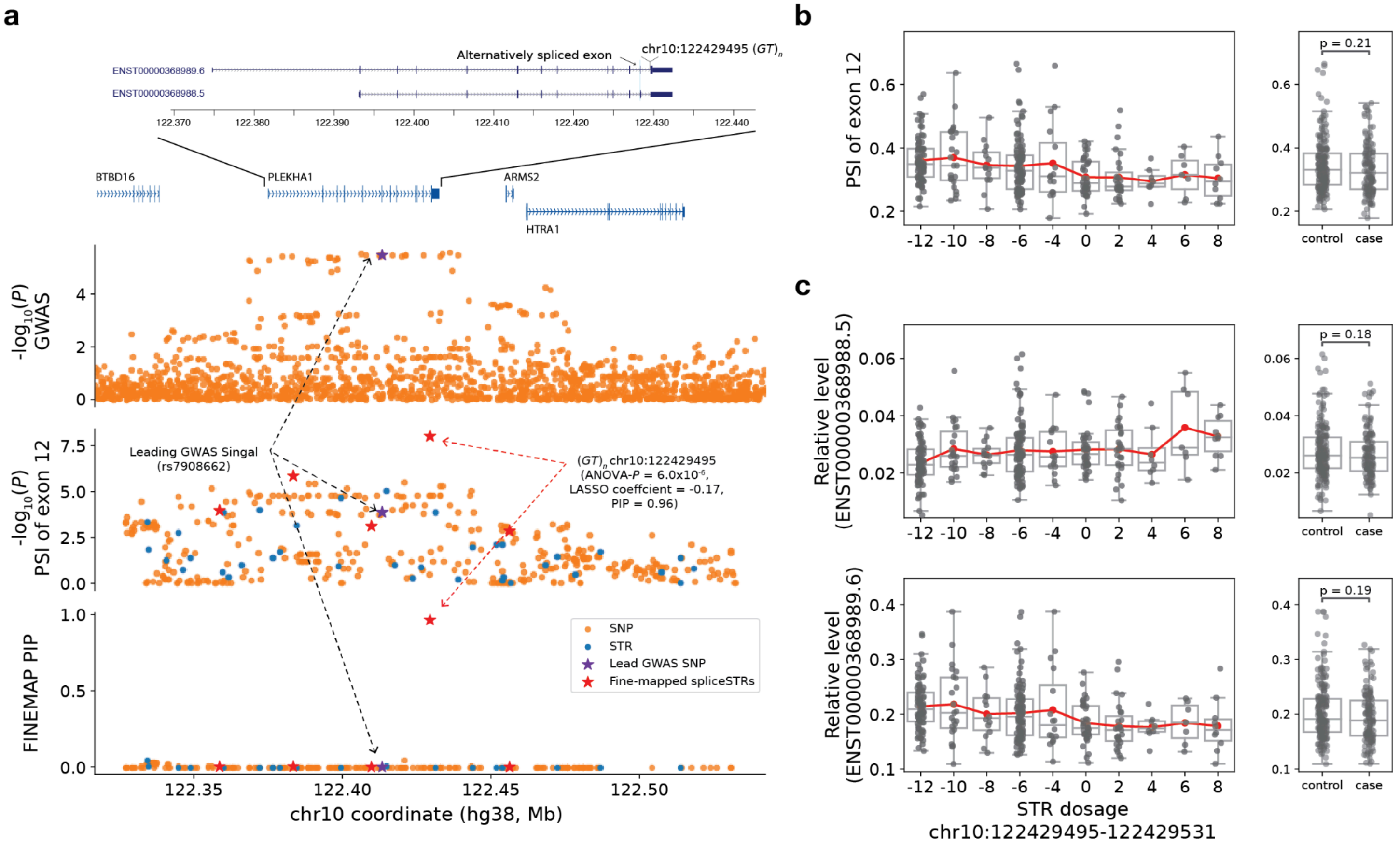

A fine-mapped spliceSTR associated with skipping of exon 12 of *PLEKHA1* colocalizes with GWAS signals for Alzheimer's disease. (a) A fine-mapped splice event that colocalizes with an Alzheimer's disease GWAS signal in *PLEKHA1*. The left panels, from top to bottom, provide genomic annotations, including a zoomed-in view to show *PLEKHA1* transcripts, -$\log_{10}$(*P*-values) for Alzheimer's disease GWAS, -$\log_{10}$(*P*-values) for splicing association tests, and FINEMAP posterior inclusion probabilities (PIPs) for all nominally significant SNPs and STRs associated with exon 12 skipping. The *x*-axis shows genomic coordinates based on the hg38 reference. (**b**) Association between STR dosage and the PSI of exon 12 of *PLEKHA1* (ENST00000368989.6). In the left panel: the x-axis represents the STR dosage (sum of repeat lengths across both chromosome copies, in bp relative to the hg38 reference) of an $(GT)_n$ STR (hg38:chr10:122429495-122429531) within an intron of *PLEKHA1*. The y-axis represents the PSI of exon 12 (ENST00000368989.6). Each dot represents a single individual. Box plots summarize the distribution of PSI values. Horizontal lines show median values, boxes span from the 25th percentile (Q1) to the 75th percentile (Q3). Whiskers extend to $Q1 - 1.5 \times IQR$ (bottom) and $Q3 + 1.5 \times IQR$ (top), where IQR is the interquartile range (Q3–Q1). The black line shows the mean expression for each x-axis value. The right panel compares the PSI of the same exon (exon 12; ENST00000368989.6) in controls (n=194) or cases (samples identified with bipolar disorder or schizophrenia, n=142). *P*-values were labeled in the plot (Mann-Whitney two-sided nominal). (**C**) Association of STR dosage vs. *PLEKHA1* isoforms. The left panel shows the relative abundance of *PLEKHA1* transcripts vs. STR dosage. The right panel compares the relative abundance of the transcripts in controls (n=194) and cases (samples identified with bipolar disorders or schizophrenia, n=142).

Supplementary Figure 20

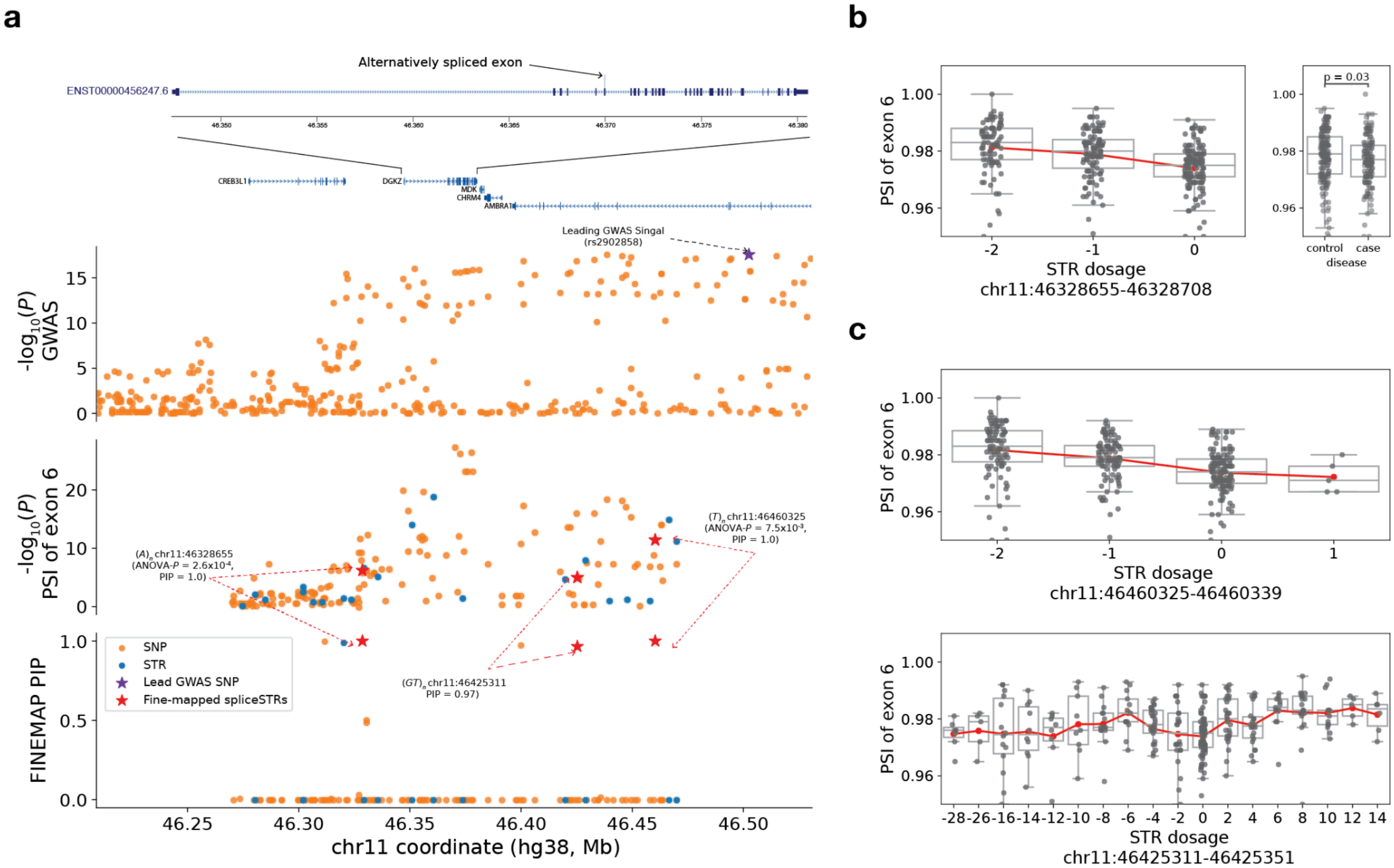

A fine-mapped spliceSTR associated with skipping of exon 6 of *DGKZ* colocalizes with GWAS signals for schizophrenia. (a) A fine-mapped splice event that colocalizes with a schizophrenia GWAS signal. The left panels, from top to bottom, provide genomic annotations, including a zoomed-in view to show *DGKZ* transcripts, -log$_{10}$(*P*-values) for schizophrenia GWAS, -log$_{10}$(*P*-values) for splicing association tests, and FINEMAP posterior inclusion probabilities (PIPs) for all nominally significant SNPs and STRs associated with exon 6 skipping of *DGKZ (*ENST00000456247.6). The *x*-axis shows genomic coordinates based on the hg38 reference. (**b**) Association between STR dosage (hg38:chr11:46328655-46328708) and the PSI of exon 6 of *DGKZ1* (ENST00000456247.6). In the left panel: the x-axis represents the STR dosage (sum of repeat lengths across both chromosome copies, in bp relative to the hg38 reference) of an $(A)_n$ STR (hg38:chr11:46328655-46328708) upstream of *DGKZ1*. The y-axis represents the PSI of exon 6 (ENST00000456247.6). Each dot represents a single individual. Box plots summarize the distribution of PSI values. Horizontal lines show median values, boxes span from the 25th percentile (Q1) to the 75th percentile (Q3). Whiskers extend to Q1 − 1.5 × IQR (bottom) and Q3 + 1.5 × IQR (top), where IQR is the interquartile range (Q3–Q1). The black line shows the mean expression for each x-axis value. The right panel compares the PSI of the same exon (exon 6; ENST00000456247.6) in controls (n=194) or cases (samples identified with bipolar disorder or schizophrenia, n=142). *P*-values were labeled in the plot (Mann-Whitney two-sided nominal). (**c**) Additional fine-mapped STRs that are associated with the PSI of exon 6 of *DGKZ1* (ENST00000456247.6).

Supplementary Figure 21

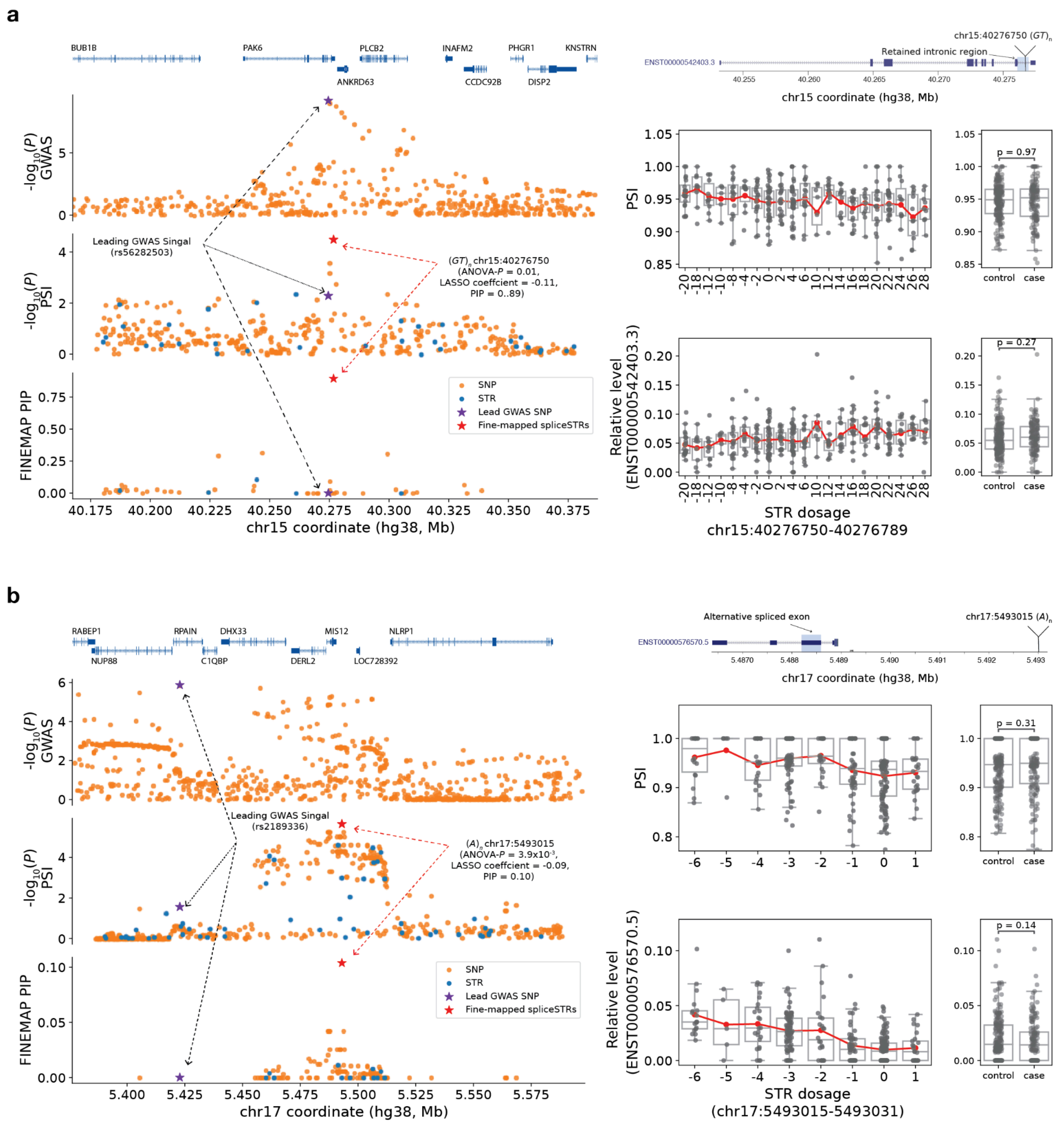

Additional examples of fine-mapped spliceSTRs that colocalized with GWAS signals. (a) A fine-mapped spliceSTR associated with an intron retention of *PAK6* colocalizes with GWAS signals for schizophrenia. The left panels, from top to bottom, provide genomic annotations, -$\log_{10}$(*P*-values) for schizophrenia GWAS, -$\log_{10}$(*P*-values) for splicing association tests, and FINEMAP posterior inclusion probabilities (PIPs) for all nominally significant SNPs and STRs associated with an intron retention event of *PAK6 (*ENST00000542403.3). The *x*-axis shows genomic coordinates based on the hg38 reference. The right panels show a zoomed-in view to show *PAK6* transcripts (top panel), including location of retained intronic region and

its fine-mapped STR, the association of STR dosage vs. the PSI of the intron retention (ENST00000542403.3, middle panel), and a *PAK6* isoform that associated with that colocalized fine-mapped spliceSTR (bottom panel), along with box plots comparing the PSI of intron retention of *PAK6,* or the relative abundance of the transcript ENST00000542403.3 in controls (n=194) and cases (samples identified with bipolar disorders or schizophrenia, n=142). *P*-values were labeled in the plot (Mann-Whitney two-sided nominal). Each dot represents a single individual. Box plots summarize the distribution of PSI values. Horizontal lines show median values, boxes span from the 25th percentile (Q1) to the 75th percentile (Q3). Whiskers extend to Q1 − 1.5 × IQR (bottom) and Q3 + 1.5 × IQR (top), where IQR is the interquartile range (Q3–Q1). The red line shows the mean expression for each x-axis value. **(b)** A fine-mapped spliceSTR associated with skipping of exon 3 of *MIS12* colocalizes with GWAS signals for schizophrenia. The left panels, from top to bottom, provide genomic annotations, -log10(*P*-values) for schizophrenia GWAS, -log10(*P*-values) for splicing association tests, and FINEMAP posterior inclusion probabilities (PIPs) for all nominally significant SNPs and STRs associated with the skipping of exon 3 of *MIS12 (*ENST00000576570.5). The *x*-axis shows genomic coordinates based on the hg38 reference. The right panels show a zoomed-in view to show *MIS12* transcripts (top panel), including location of exon 3 and its fine-mapped STR, the association of STR dosage vs. the PSI of the skipping of exon 3 of *MIS12* (ENST00000576570.5, middle panel), and a *MIS12* isoform that associated with that colocalized fine-mapped spliceSTR (bottom panel), along with box plots comparing the PSI of intron retention of *PAK6,* or the relative abundance of the transcript ENST00000576570.5 in controls (n=194) and cases (samples identified with bipolar disorders or schizophrenia, n=142).

Supplementary References

1. Sehgal, A., Ziaei Jam, H., Shen, A. & Gymrek, M. Genome-wide detection of somatic mosaicism at short tandem repeats. *Bioinformatics* **40**, btae485 (2024).
2. Hamanaka, K. *et al.* Genome-wide identification of tandem repeats associated with splicing variation across 49 tissues in humans. *Genome Research* **33**, 435 (2023).
3. Cui, Y. *et al.* Multi-omic quantitative trait loci link tandem repeat size variation to gene regulation in human brain. *Nature Genetics* **57**, 369–378 (2025).
4. Skipper analysis of eCLIP datasets enables sensitive detection of constrained translation factor binding sites. *Cell Genomics* **3**, 100317 (2023).
